# Ultra-long-working-distance spectroscopy of single nanostructures with aspherical solid immersion microlenses

**DOI:** 10.1038/s41377-020-0284-1

**Published:** 2020-03-27

**Authors:** Aleksander Bogucki, Łukasz Zinkiewicz, Magdalena Grzeszczyk, Wojciech Pacuski, Karol Nogajewski, Tomasz Kazimierczuk, Aleksander Rodek, Jan Suffczyński, Kenji Watanabe, Takashi Taniguchi, Piotr Wasylczyk, Marek Potemski, Piotr Kossacki

**Affiliations:** 1 Faculty of Physics, University of Warsaw, ul. Pasteura 5, 02-093 Warsaw, Poland; 2 National Institute for Materials Science, Tsukuba, Ibaraki 305-0044 Japan; 3 Laboratoire National des Champs Magnétiques Intenses, CNRS-UJF-UPS-INSA, avenue des Martyrs 25, 38042 Grenoble, France

**Keywords:** Optical spectroscopy, Applied optics, Integrated optics, Micro-optics, Optical materials and structures

## Abstract

In light science and applications, equally important roles are played by efficient light emitters/detectors and by the optical elements responsible for light extraction and delivery. The latter should be simple, cost effective, broadband, versatile and compatible with other components of widely desired micro-optical systems. Ideally, they should also operate without high-numerical-aperture optics. Here, we demonstrate that all these requirements can be met with elliptical microlenses 3D printed on top of light emitters. Importantly, the microlenses we propose readily form the collected light into an ultra-low divergence beam (half-angle divergence below 1°) perfectly suited for ultra-long-working-distance optical measurements (600 mm with a 1-inch collection lens), which are not accessible to date with other spectroscopic techniques. Our microlenses can be fabricated on a wide variety of samples, including semiconductor quantum dots and fragile van der Waals heterostructures made of novel two-dimensional materials, such as monolayer and few-layer transition metal dichalcogenides.

## Ultra-long-working-distance spectroscopy of nanostructures with elliptical 3D-printed microlenses

Tiny elliptical microlenses efficiently focus light onto and extract it from nano-sized light emitters, enabling an ultra-long-working-distance measurements. Aleksander Bogucki from the Faculty of Physics, University of Warsaw and colleagues tested their microlenses by 3D printing them on top of light-emitting quantum dots and a more fragile layered structure made of novel 2D materials. They demonstrated the microlenses can be deterministically placed onto specific parts of a sample, or can be printed as a large-scale array of hundreds of lenses on samples containing a large number of light emitters. It can also be printed onto fragile materials without affecting their properties. The findings could pave the way for their use in a wide range of applications, including single-nanowire lasers, photoconductive antennas, optical setups in high magnetic fields, and in integrated photonic circuits.

## Introduction

Efficient light delivery to and collection from micro-optical systems, in particular light emitters, is of paramount importance for their application potential and performance. It has been approached in many different ways: by placing mirrors beneath the light emitters, coating the substrate surface with anti-reflective layers to reduce internal reflection or shaping the transparent casing into the form of lenses, mesas, gratings or nanowires^1–10^. These approaches help increase the critical angle of total internal reflection and/or reduce the Fresnel reflections at the interface. In the case of semiconductor nanostructures, solutions relying on solid immersion lenses (SILs) fabricated on top of the emitter, typically in the shape of a hemisphere, are frequently used. SILs manufactured with 3D subtractive techniques, such as electron beam lithography or focused ion beam, can increase the photon extraction up to 23% or even further up to 40% by employing additional optical structures^7,11^. None of these methods, however, have turned out to be fully satisfactory and free from considerable disadvantages: they still require additional light-collection optics of a high numerical aperture or cannot be used with atomically thin semiconductors, such as transition metal dichalcogenides. Therefore, efficient light collection from micro/nanoemitters and beam shaping (collimation) still present challenges in photonics and optical nanotechnology.

**Fig. 1:**
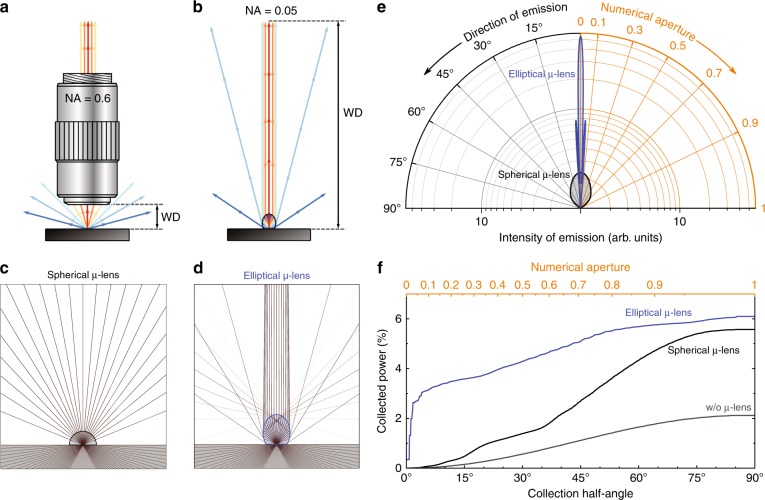
Superior efficiency of light collection with an elliptical µ-lens compared to the situation with a hemispherical lens for low-numerical-aperture collection optics. **a** Standard experimental setup for optical study of light emitters. The microscope objective limits both the working distance (WD) and the aperture. **b** Schematic diagram showing the desired performance of a single nonspherical µ-lens replacing the objective and forming the emitted light into a low-divergence beam. As a result, the outgoing beam can be collected by a low-numerical-aperture setup (e.g., a spectrometer). **c** and **d** Results of ray-tracing simulations showing the difference in the beam shape achievable with spherical and elliptical µ-lenses. Both lenses are supposedly made of materials with refractive index *n* = 1.53 and are deposited on a high-refractive-index (*n* = 3) substrate containing a point light source located 50 nm below its surface. **e** 2D slices of the far-field radiation patterns for the hemispherical (black) and elliptical (blue) µ-lenses, calculated with finite-difference time-domain (FDTD) software. The spherical µ-lens has a close-to-isotropic far-field pattern, whereas the elliptical µ-lens exhibits high directivity of light emission. A logarithmic scale is used in the radial direction for clarity. **f** Total power of the collected light as a function of the collection half-angle. The advantage of the elliptical µ-lens (blue curve) over the spherical one (black curve) is particularly visible for collection half-angles below 40° (NA<0.65)—for low-NA setups, the power collected with the elliptical µ-lenses substantially exceeds the amount of light that can be accumulated with traditional spherical µ-lenses. The collection efficiency without any lens (grey curve) is strongly limited due to the total internal reflection

Once the light is extracted from the emitter, it must be shaped into a beam and directed to a measurement setup or another unit in an integrated electro-optical circuit. For industrial millimetre-scale light-emitting devices, such as LEDs, this problem has many well-established solutions, e.g., free-form lenses or dielectric total internal reflecting concentrators (DTIRCs)^12–14^. On the other hand, for prototype nanoscale emitters, high-numerical-aperture (NA) objectives (see Fig. 1a) are commonly used, resulting in a trade-off between the brightness and working distance (WD). In many experiments, however, the lens cannot be set close enough to the sample, and a significant amount of light is lost. These experiments, in particular, include those at cryogenic temperatures, in high continuous or pulsed magnetic fields, and with microwave or terahertz radiation as well as efficient light coupling into fibres for photonic lab-on-chip applications.

Surprisingly, the issues of light extraction and beam forming for nanoscale emitters are usually still treated separately, even though SILs are frequently manufactured by direct laser writing (DLW)—a versatile technique capable of printing lenses of virtually any shape. To our knowledge, the only approach to date for simultaneously addressing both problems for nanoscale point emitters has been to employ an evolutionary algorithm for light extraction from a nanowire, designed specifically to minimise the footprint of the structure^15^.

In this article, we present broadband elliptical microlenses (µ-lenses) fabricated by DLW, which enable spectroscopic measurements of single light emitters with extremely low-NA collection optics (see Fig. 1b). In a standard realisation of DLW-printed µ-lenses, the observed increase in the light extraction efficiency is due to a reduction of the Fresnel reflections and an increase in the critical angle of total internal reflection^16,17^. Here, in addition to doubling the number of photons collected from the sample, the use of a nonspherical µ-lens results in forming the collected light into an ultra-low-divergence beam (measured beam divergence half-angle smaller than 1°). Thus, the emitted light can be directly introduced into the collection optics with an effective WD of ~600–700 mm, which is 70 times longer than that of standard high-NA long-WD microscope objectives.

We demonstrate the performance of our µ-lenses for two types of semiconductor emitters: (i) heterostructures based on monolayer transition metal dichalcogenides and hexagonal boron nitride (h-BN) and (ii) self-assembled quantum dots (QDs). The µ-lens design works well for a broad spectral range spanning the visible and near-infrared bands.

## Results

### Optimum lens shape design

Thus far, the simplest and most common form of the µ-lens has been a hemisphere placed above a nanoscale light emitter^2,16–21^. Usually, it is made of a material with a refractive index between that of the surrounding medium (low) and that of the sample (high), which not only enables an increase in the critical angle of total internal reflection but also significantly reduces the losses caused by the Fresnel reflections. The distribution of light leaving the hemispherical lens is isotropic (see Fig. 1c), and a microscope objective with a high NA is still required for efficient light collection.

Ideally, the light collection structure should (i) minimise the Fresnel reflections, (ii) be able to send the outgoing light towards distant collection optics and (iii) allow for fabrication on any sample/substrate. To achieve this with a single lens, it has to transform the homogeneous angular distribution of the photoluminescence (PL) from a point emitter into a collimated beam (see Fig. 1d). Within the approximation of geometric optics, the optimal lens shape when the refractive indices of the lens and substrate match is a Cartesian oval—an ellipsoid of revolution, known for almost four centuries^22^. To obtain a collimated beam, the emitter should be placed at the focal point situated further from the upper apex. The relation between the shorter half-axis *a* of the ellipsoid and the longer half-axis *b* (see Supplementary Information) is given by

1 $$b = \frac{{an_1}}{{\sqrt {n_1^2 - n_0^2} }}$$

where *n*_0_ is the refractive index of the surrounding medium (air) and *n*_1_ is the refractive index of the material the ellipsoid is made of (in our case, 1.53) ^23^.

**Fig. 2:**
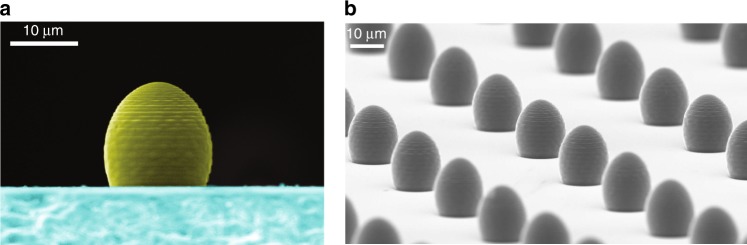
Scanning electron microscope (SEM) images of 3D-printed µ-lenses. **a** Side view of a µ-lens (coloured in yellow) fabricated on a glass substrate (coloured in blue). Regardless of the residual roughness of the produced structures, the presented optical quality is more than sufficient for obtaining a well-collimated light beam. **b** Series of µ-lenses distributed in a regular way across a substrate containing randomly dispersed light emitters, e.g., quantum dots. Due to the short printing time, it is possible to produce hundreds of µ-lenses on the substrate surface and search for interesting light emitters afterwards. A regular grid of lenses also provides a convenient coordinate system for marking the positions of individual light sources. This enables moving the sample with a selected emitter to a different laboratory and measuring the same emitter in various experimental setups

In general, the emitter may be embedded in a high-refractive-index substrate (*n*_2_) and located at a distance *d* below its surface. To a first (paraxial) approximation, for *d* << *b*, the lens still takes on the form of an elongated spheroid, cut on the bottom side by the substrate, while the presence of the substrate defines the apparent position of the emitter according to Snell’s law (see Fig. 2a). In this case, the lens height (the distance between the ellipsoid top and the substrate surface) is given by

2 $$h = a\sqrt {\frac{{n_1 + n_0}}{{n_1 - n_0}}} - d\frac{{n_1}}{{n_2}}.$$

It is worth noting that under these conditions, the µ-lens centre is no longer positioned at the substrate surface. This simple modification combined with a change of the well-established hemispherical design of a lens into an elliptical one offers a solution for both light extraction from the sample and light redirection towards the collection optics.

The above approach works well in the limit of geometric optics and allows for an arbitrary size of a µ-lens. However, to obtain a beam with the lowest possible divergence, the analysis must be done in the framework of wave optics^24,25^. We have approached this by solving Maxwell’s equations with a finite-difference time-domain (FDTD) method. Here, the maximum achievable WD for a given NA is determined by the minimal diameter of the µ-lens—the smaller the lens diameter is, the larger the divergence of the outgoing beam. For a 1-inch lens placed ~0.5 m from the sample, the minimum diameter of a µ-lens should be ~15 µm.

Figure 1e shows the simulated intensity of light emitted at different angles from a point source located 50 nm below the surface of a high-refractive-index (*n* = 3) semiconductor. The distribution of light emitted from an elliptical lens is highly directional (blue curve), in contrast to that from a hemispherical lens (black curve), which is close to isotropic. Figure 1f presents the integrated light intensity as a function of the collection angle. The advantage of the elliptical shape over the spherical shape is particularly visible for collection angles below 40° (NA < 0.65). For small numerical apertures (NA < 0.1), the increase in the intensity exceeds two orders of magnitude.

### Lens fabrication

µ-lenses were produced with two-photon 3D photolithography (DLW), which has been proven to yield high-quality optical elements of arbitrary shape for lab-on-chip and optical fibre applications^26–30^. Figure 2a shows a scanning electron microscope (SEM) image of an elliptical µ-lens with a diameter of 15 µm. Owing to the short printing time (<4 min for a standard 3D lithography system with piezoelectric stages; see the “Methods” section for details), it is possible to manufacture hundreds of lenses, each covering a small portion of the sample surface, and to search for the emitters of interest afterwards. A grid of lenses (see Fig. 2b) provides a coordinate system for recording the emitter positions. This nondeterministic approach significantly simplifies the manufacturing procedure. Alternatively, high-accuracy deterministic positioning of µ-lenses can be performed when the location of an emitter of interest is known or the emitter is visible under an optical microscope^7,16^.

### Applications in spectroscopy

To demonstrate the performance of the µ-lenses, we tested them for two different semiconductor systems: self-assembled QDs and van der Waals heterostructures made of novel quasi-two-dimensional materials, i.e., monolayers of semiconducting transition metal dichalcogenides (S-TMDs) and h-BN. From the viewpoint of DLW-based 3D printing, these represent two very distant areas in the field of solid-state physics and technology.

The QDs, which are nanometre-sized structures formed during epitaxial growth of lattice-mismatched materials, are typically buried under a capping layer that insulates them from the detrimental influence of oxygen and water vapour in the air. Moreover, they are firmly linked to the surrounding host material. For the S-TMD-based van der Waals heterostructures, which are typically weakly bound to the supporting substrate, contact with just the ambient atmosphere and the contaminants it contains, as well as with many other chemicals can destroy them or irreversibly alter their properties. As a result, 3D printing of µ-lenses over such objects constitutes a real challenge that, to the best of our knowledge, has not been undertaken thus far.

**Fig. 3:**
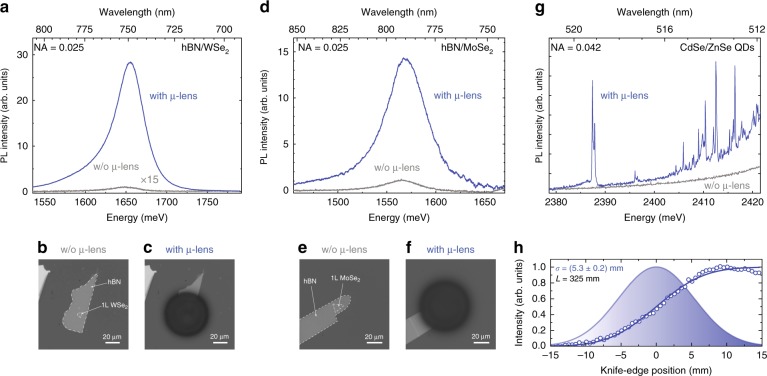
Improved directivity of light emitted by different types of nano- and microscale emitters equipped with elliptical µ-lenses. **a** Photoluminescence spectra collected in a low-NA setup from an h-BN/1 monolayer WSe_2_ heterostructure before (grey curve) and after (blue curve) printing the µ-lens. A strong increase (by ×450) in the collected light intensity is observed after fabrication of the µ-lens on top of the heterostructure. **b**, **c** Optical microscope images of the h-BN/1 monolayer WSe_2_ heterostructure before and after 3D printing of the µ-lens. **d**–**f** Similar results obtained for a different S-TMD material: monolayer MoSe_2_. **g** Effect of elliptical µ-lenses on the performance of other nanoscale emitters—semiconductor quantum dots (QDs). As shown, the excitonic emission lines of single CdSe/ZnSe QDs can be distinguished without the help of an additional microscope objective. **h** QD photoluminescence beam size measured with the travelling-knife-edge technique. The integrated photoluminescence intensity (open circles), error function fitted to the experimental data (blue curve) and corresponding Gaussian distribution (blue shaded shape) show a 16.4 milliradian (*θ*=0.94°) beam divergence (NA=0.0164). The knife-edge was placed at *L*=325 mm from the sample. The PL spectra were measured at room temperature for the h-BN-capped S-TMD flakes and at 1.6 K for the QDs

The interest in monolayer and few-layer S-TMDs and other 2D materials results from their unique electronic and optical properties^31^ as well as their mutual compatibility, which opens up the possibility of stacking them to form artificial crystals with tailored properties^32^. The S-TMDs, with a non-zero bandgap, are particularly well suited for optical applications, including optoelectronic sensors and light emitters^33–38^. We decided to use two popular representatives of the S-TMD family, namely, monolayer WSe_2_ and monolayer MoSe_2_. We demonstrate that additive DLW of polymer structures on top of S-TMD-based and h-BN-based heterostructures enhances the collection efficiency without deteriorating their optical properties. Figure 3b, c, e, f show optical microscope images of heterostructures consisting of a single layer of WSe_2_ or MoSe_2_ covered with a thin flake of h-BN deposited on a piece of a 90 nm SiO_2_/Si substrate before and after printing the µ-lenses. The characteristic lateral dimensions of the S-TMD monolayers are below 10 µm to ensure that they can be approximated as point-like light sources. Figure 3a, d display room-temperature PL spectra collected without an external microscope objective (see the “Methods” section for details) for the h-BN/WSe_2_ and h-BN/MoSe_2_ heterostructures without (grey curve) and with (blue curve) the elliptical µ-lenses. Both spectra were acquired with a lens of *f* = 500 mm and a 1-inch diameter. The enhancement of the collection efficiency with the elliptical µ-lens reaches ×450 for the h-BN/WSe_2_ heterostructure, while for the h-BN/MoSe_2_ heterostructure, the enhancement is approximately ×15. The difference between these numbers can be explained by the fact that the dimensions of the monolayer flakes of WSe_2_ and MoSe_2_ are not the same, while the diameter of the µ-lens is fixed at 70 µm. Since the WSe_2_ monolayer is a few times smaller than the MoSe_2_ monolayer, it better satisfies the approximation of the point-like emitter, for which the elliptical µ-lens was designed.

The second test structure contains self-assembled QDs, which attract interest due to their remarkable optical properties, such as single photon emission or entangled photon pair generation^1,2,4,11^. This system is a better model of a point-like light source. In contrast to the h-BN/S-TMD heterostructures, where single µ-lenses were fabricated in specific areas determined by the heterostructure locations, here, we produced a large-scale array of 1400 µ-lenses (each 15 µm in diameter) on a sample containing either CdSe/ZnSe QDs emitting in the 500–520 nm range or CdTe/ZnTe QDs emitting in the 600–650 nm range. All QDs are located 50 nm below the sample surface (more details are given in the Supplementary Information).

Figure 3g shows PL spectra of CdSe/ZnSe QDs collected without a microscope objective from a sample without a µ-lens (grey curve) and with an elliptical µ-lens (blue curve). The spectrum measured without the µ-lens does not show any individual emission lines. Under the excitation laser spot (28 µm in diameter), there are ~10^4^ QDs emitting at different wavelengths. As a result, many individual sharp lines merge into a nearly uniform PL spectrum.

On the other hand, the laser beam impinging on the elliptical µ-lens is focused to a spot with a diameter below 1 µm (close to the diffraction-limited size) and thus excites much fewer QDs. Moreover, among all the excited QDs, only those satisfying the collimation conditions efficiently contribute to the collected PL spectrum, and sharp emission lines of individual QDs are clearly visible. The intensity gain in this case is estimated to be approximately 100 (see the “Methods” section for details).

**Fig. 4:**
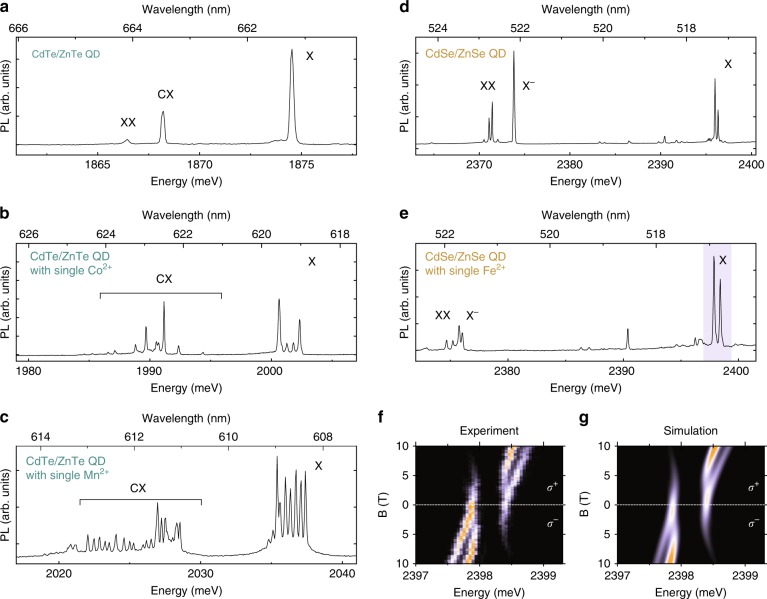
Benefits of using elliptical µ-lenses in the photoluminescence study of single QDs. In both telluride-based and selenide-based QD systems, the use of elliptical µ-lenses enables observation of sharp emission lines without the help of an additional microscope objective. **a** Photoluminescence (PL) spectrum of a single CdTe/ZnTe QD without a magnetic ion. **b** and **c** PL spectra of CdTe/ZnTe QDs with single cobalt 2+ and manganese 2+ ions, respectively. Characteristic splitting of the exciton (X) and charged exciton (CX) lines is clearly visible. **d** PL spectrum of a single CdSe/ZnSe QD without a magnetic ion. **e** PL spectrum of a single CdSe/ZnSe QD with a single iron 2+ ion. **f** Magnetic-field evolution of the excitonic emission line (X) of a QD with a single Fe^2+^ ion shown in panel **e** (the analysed spectral region is marked in **e** by the blue box). The colour coding corresponds to the PL intensity expressed in arbitrary units. **g** Modelling of the experimental data presented in **f**. A comparison between the measured and simulated data clearly indicates that the µ-lens does not influence the circular polarisation of the emitted light

We note that manufacturing µ-lenses over the QDs did not visibly affect their properties. Figure 4a, d show the PL spectra of a single CdTe/ZnTe QD and a single CdSe/ZnSe QD coupled to a µ-lens. The spectra feature well-resolved emission lines corresponding to recombination of a neutral exciton (X), a charged exciton (CX) and a biexciton (XX), consistent with the emission of QDs in unstructured samples^39,40^.

### Beam shape characterisation

To measure the resulting PL beam size and divergence, we used the travelling knife-edge technique^41^. In Fig. 3h the total PL intensity of CdSe/ZnSe QDs with a µ-lens printed immediately above them is shown for different positions of the knife edge (empty circles). The distance between the sample and the knife edge was set to *L* = 325 mm. The blue curve represents the result of fitting an error function to the experimental data and indicates a close-to-Gaussian beam profile. A corresponding Gaussian function with a width of *σ* = (5.3 ± 0.2) mm is drawn in the background as the blue shaded area. This result indicates that the half-angle divergence of the PL beam is 16.4 milliradians (*θ* = 0.94°), which means that the light emitted from the sample can be collected by an optical setup with an NA as low as 0.016.

### Microlens arrays for solotronic systems

The CdTe/ZnTe and CdSe/ZnSe QD systems were selected to demonstrate the application of the elliptical µ-lenses in the field of optoelectronics based on solitary dopant—solotronics^42,43^. When doped with single magnetic ions, such QDs can be used to efficiently probe and manipulate the spin of the magnetic ion they contain^44–46^, which otherwise is not optically active. A major difficulty in the study of this type of system is the nondeterministic character of self-assembled growth.

We fabricated regular arrays of µ-lenses on a series of samples with QDs doped with various magnetic ions. In each case, we were able to find a µ-lens printed over a singly doped QD, including a CdTe/ZnTe QD with a single Co^2+^ ion (Fig. 4b), a CdTe/ZnTe QD with a single Mn^2+^ ion (Fig. 4c), and a CdSe/ZnSe QD with a single Fe^2+^ ion (Fig. 4e). For all of them, we clearly observed a characteristic splitting of the exciton line into components corresponding to different spin projections onto the quantisation axis^42–44^. In particular, Fig. 4f displays the magnetic-field evolution of the excitonic emission lines highlighted in Fig. 4e by the blue box. It features a characteristic zero-field anticrossing and shows a gradual rise of the polarisation degree due to thermalisation occurring with increasing magnetic field^42^. The results of numerical simulations are shown in Fig. 4g (for simulation details, see Supplementary Information). The direct correspondence between the experimental and simulated data indicates that the use of µ-lenses does not affect the polarisation of the emitted light or its temporal stability.

## Discussion

We demonstrated that elliptical µ-lenses can work over a broad spectral range—at least from 500 to 800 nm—and potentially also at longer wavelengths since the IP-Dip photoresist we used is transparent up to 1610 nm^26^. We showed that DLW-printed microstructures can be produced not only over optically active objects well protected against environmental conditions (such as QDs) but also on extremely fragile S-TMD-based and h-BN-based heterostructures, whose internal cohesion and adhesion to the substrate are governed by weak, dipole–dipole-like van der Waals interactions.

### Perspectives

Owing to the broad compatibility of the DLW technique, the designed elliptical µ-lenses can be directly applied to a variety of optical systems. An example of such a system potentially benefiting from the µ-lenses is a single-nanowire laser^47,48^. Usually, after the growth process, nanowires are transferred onto a substrate with their longer axis parallel to the substrate surface. Most of the light generated by these lasers is emitted from the point-like nanowire ends, and as a result, one obtains an isotropic far-field pattern. The application of the elliptical µ-lenses could result in this case in an ultra-low-divergence laser beam.

Nanowires also serve as effective detectors of terahertz radiation and as such are used in photoconductive antennas (PCAs)^49^. A device of this type requires pulsed laser illumination focused on the nanowire to generate a sizeable electrical signal. The terahertz radiation is brought to the antenna by a parabolic mirror, and the infrared laser feed is usually provided through a few millimetres in diameter hole in the centre of the mirror. Unfortunately, due to the low NA of the infrared part of the setup, the laser power must be inconveniently high. This issue can be readily solved by using an elliptical µ-lens, enabling the use of standard focusing optics with a small NA to obtain a preliminary focal spot of the infrared laser, which can then be further focused to the single micrometre level.

The limited NA of an optical setup becomes problematic in experiments performed in high magnetic fields where access to the sample is restricted. Moreover, due to inductance issues, one has to avoid metal parts being close to the sample. Similar limitations play an important role in the case of measurements involving microwave radiation, e.g., optically detected magnetic resonance (ODMR)^50,51^ or optically detected nuclear resonance^52^. As microwave cavities are inconvenient for optical measurements of single nanoscale emitters, an elliptical µ-lens could help one focus on the emitter, even with a poorly focused excitation beam, and simultaneously increase the amount of light collected by distant optical elements.

It is worth noting that potential applications of the elliptical µ-lenses are not limited to samples that emit light. Powerful optical techniques, such as Raman scattering, can also benefit from the results presented in this paper. In measurements of this type, the delivery of excitation laser light with sufficient power density to the sample surface is as important as efficient collection of the scattered light, whose intensity is typically very low. Again, as Raman scattering is usually isotropic, utilisation of the elliptical µ-lenses can increase the available WD and substantially decrease the required NA of the collection optics. It is important to note that the Raman signal of the µ-lens itself should not obscure the signal from the sample. The photoresist used in this article (IP-Dip Nanoscribe GmbH) does not exhibit any Raman scattering response in two spectral regions: 1800–2850 cm^−1^ and above 3115 cm^−1^. This does not mean, however, that other parts of the electromagnetic spectrum are totally inaccessible. One must keep in mind that since the highest intensity of the laser light is concentrated on the sample surface and not inside the µ-lens volume, the Raman scattering signal from the photoresist is relatively weak (see Supplementary Information for details) and does not significantly affect the Raman spectrum of the sample.

A combination of the above functionalities offered by the elliptical µ-lenses might also be useful for integrated photonic and microfluidic circuits, e.g., opto-fluidic chips^53,54^. The design and fabrication of these devices have recently become remarkably advanced^55,56^. Their proper operation requires focusing a single or multiple laser beams with micrometre precision and collecting the emitted optical signal. With the elliptical µ-lenses, one could use small-NA and long-WD optics to simultaneously achieve both of these goals.

Finally, the elliptical µ-lenses can be used in devices or circuits based on optical fibres, which are still the most common means of introducing light to and collecting it from integrated chips. Such lenses can actually serve as effective coupling elements after minor modification of their design. If the centre of the lens ellipsoid is shifted away from the sample surface (see Supplementary Information), the beam is focused at a few tens of micrometres above the sample. The width of the focal spot can be precisely tuned so that a good match between the distribution of light leaving the ellipsoid and the field mode of an optical fibre can be achieved. Such a modified µ-lens combined with a DLW-printed optical fibre microconnector^57^ can be directly implemented on an integrated chip.

We have shown that elliptical µ-lenses, apart from boosting the efficiency of light extraction from and delivery to single quantum emitters, shape the PL light into an ultra-low-divergence beam. Unlike previous approaches, the µ-lenses are capable of collecting the emitted light without the use of additional high-NA objectives. We have demonstrated the µ-lens compatibility with a variety of semiconductor light-emitting systems, including monolayers of transition metal dichalcogenides and self-assembled QDs. We have shown that 3D DLW-based fabrication of micro- and nanostructures on fragile van der Waals heterostructures is possible. Two µ-lens printing strategies have been demonstrated: (i) deterministic, for when the objects to be equipped with µ-lenses are well visible or their locations in/on the substrate are precisely known, and (ii) nondeterministic, suitable when the substrate contains a large number of randomly distributed light emitters, among which only some possess desirable properties (such as a single magnetic ion inside a QD). Our results pave the way for new ultra-long-working-distance optical measurements of micro- and nano-objects not achievable thus far with a standard spectroscopy technique requiring bulky, high-NA objectives.

## Materials and methods

### Microlens fabrication

Microlenses were fabricated by 3D additive DLW using a two photon photolithography workstation (Photonic Professional, Nanoscribe GmbH). A near-infrared femtosecond laser beam was focused inside a droplet of UV-curable photoresist, acting simultaneously as an immersion medium for a microscope objective (dip-in laser lithography—DiLL). Only inside the focal spot volume was the intensity of the laser light high enough for two-photon polymerisation (TPP) to occur. The smallest possible volume to be solidified, called a voxel, was an ellipsoid of revolution of size ~0.4 µm in the plane perpendicular to the laser beam and 1.2 µm along the beam. When fabricating the elliptical µ-lenses, we used a ×100, NA = 1.3 (Zeiss) immersion objective and a negative-tone photoresist (IP-Dip, Nanoscribe GmbH). The relative position of a voxel with respect to the sample was changed with the aid of a piezoelectric XYZ stage. After the exposure, the resist was developed for 20 min in a bath of propylene glycol methyl ether acetate (PGMEA) stirred at 150 rpm, then washed for 20 min in isopropanol (IPA) stirred at 150 rpm and finally gently rinsed with fresh isopropanol.

To speed up the DLW printing, the trajectory of the laser inside the resist was programmed manually without any slicing software. The lens was printed layer by layer, and the voxels followed a spiral path in the clockwise and counterclockwise directions alternately. To preserve a smooth shape of the ellipsoid, a three-fold exposure of its outer shell with decreased laser power was performed, and the vertical spacing between the layers was adjusted to the local curvature of the lens.

The choice of IP-Dip resist was dictated by the fact that it maintains its mechanical and optical properties down to cryogenic temperatures^57^. Other resists, however, with different optical and mechanical properties can also be used in the DLW process^58,59^. The PL data along with the transmission and Raman scattering spectra of the IP-Dip (Nanoscribe GmbH) negative-tone photoresist are presented in the Supplementary Information. The details of the µ-lens printing procedure are also given there.

### Samples

Single layers of MoSe_2_ and WSe_2_ (bulk crystals purchased from HQ Graphene) were mechanically exfoliated with Microworld F07 tape and transferred by all-dry viscoelastic stamping^60^ onto SiO_2_ (90 nm)/Si substrates. After that, all samples were covered with exfoliated, 3–30 nm-thick h-BN flakes acting as a protective layer. The samples were annealed at 180 °C for 20 min immediately after the transfer to reduce the amount of air bubbles formed between the constituent layers. Capping the samples with h-BN was necessary to prevent their degradation during the photoresist development. However, there are photoresists that do not influence the quality of S-TMD samples^17,61^. The assembled van der Waals heterostructures were additionally annealed in air for 1 h at 180 °C just before the 3D printing.

Samples with self-assembled QDs were prepared by a molecular beam epitaxy (MBE) technique. More details on the growth procedures of CdTe/ZnSe and CdTe/ZnTe QDs with and without magnetic ions can be found in refs. ^40,42,43^.

### PL measurements

PL measurements were performed with diode lasers of three different wavelengths: *λ* = 405 nm for CdSe/ZnSe samples, *λ* = 532 nm for CdTe/ZnTe samples and *λ* = 647 nm for S-TMD samples. While measuring the enhancement of the PL intensity for the S-TMD-based heterostructures, the power of the laser beam focused on the sample by a single lens with a 1-inch diameter and *f* = 500 mm (without any additional microscope objectives) was kept at 24 µW. The emitted light was dispersed in a 75 cm spectrometer (Acton SP2750) equipped with a charge-coupled device (CCD) camera. In each measurement, two PL spectra were taken with the same acquisition parameters: a background spectrum from a spot on the sample where there was only substrate without any light emitter and a measured spectrum from a location where a microlens was printed over the light emitter. The final spectrum was obtained as the difference between the measured spectrum and the background spectrum. During PL measurements of single QDs, the samples were placed in an optical cryostat featuring a 10 T superconducting magnet. These measurements were performed in a pumped helium bath (1.6 K) with standard polarisation optics in the detection path.

The evaluation of the PL intensity enhancement for the QDs required taking into account the fact that without a µ-lens, the observed PL spectrum, which seemingly did not feature any narrow peaks, was in fact composed of many sharp emission lines coming from individual QDs within the perimeter of the excitation laser spot. Therefore, the PL intensity gain could be estimated as $G = I_{1{\mathrm {QD}}}^{\Delta \lambda }\left( {I_{1{\mathrm {QD}}}^{\Delta \lambda }} \right)^{ - 1}N_{{\mathrm {QDs}}}^{\Delta \lambda }$, where *I*^∆*λ*^_1QD_ is the intensity of a sharp emission line coming from a given QD under the elliptical µ-lens integrated over the spectral range ∆*λ*, *I*^∆*λ*^_QDs_ corresponds to the PL intensity obtained in the same location on the sample integrated over the same spectral range ∆*λ* but without the µ-lens, and finally, *N*^∆*λ*^_QDs_ represents the number of QDs contributing to the PL spectrum in the spectral range ∆*λ*. *N*^∆*λ*^_QDs_ can be expressed as *ρ*_QDs_*AI*^∆*λ*^_QDs_ (*I*^tot^_QDs_)^−1^, where *ρ*_QDs_ is the lateral density of QDs, equal in our case to approximately 10^9^–10^10^ cm^−2^, *A* is the area of the laser spot, and *I*^tot^_QDs_ represents the intensity of the QD PL spectrum acquired without the µ-lens integrated over the whole spectral range. The size of the excitation beam spot at the sample position was measured with a Coherent BeamMaster profiler. The value of the PL intensity gain factor *G* obtained in the course of this analysis was on the order of 100.

### Travelling knife-edge measurements

To perform travelling knife-edge measurements of the PL beam size and divergence, a motorised knife-edge was placed 325 mm from the sample. The outgoing light was introduced into a 50 µm-diameter step-index multimode optical fibre, whose NA was equal to 0.22, with the aid of a lens of *f* = 200 mm and diameter *d* = 70 mm positioned 350 mm from the sample. After filtering the laser light out with a 488 nm long-pass dichroic filter, the outgoing light was fed into a 75 cm spectrometer (Acton SP2750) equipped with a CCD camera. A diffuser plate with Lambertian scattering characteristics was placed in front of the optical fibre surface to guarantee the same intensity of light arriving at different angles. The excitation laser beam was introduced onto the optical axis with a long-pass, 2-inch × 2-inch dichroic filter placed 175 mm from the sample. To measure the NA of the experimental setup, a VRC1 detection card from Thorlabs acting as an isotropic light source was used in place of the sample. The acceptance angle of the setup was only limited by the diameter of the collection lens and was approximately five times the measured divergence of the PL beam coming from the printed µ-lens.

### Numerical simulations

The elliptical µ-lens performance was simulated within the formalism of the FDTD method for solving Maxwell’s equations with the FDTD Solutions software provided by Lumerical Inc. The 3D simulation box contained a point dipole light source (*λ* = 633 nm) positioned 50 nm below the surface of a substrate made of an *n* = 3 material and capped with an ellipsoid of revolution made of a material with *n* = 1.53. The lengths of the ellipsoid half-axes and the position with respect to the substrate surface are given by Eqs. (1) and (2). The simulation box (20 × 20 × 21 µm^3^) consisted of more than 2.6 × 10^8^ Yee cells and was limited by perfectly matched boundary layers at the top and bottom and by layers satisfying symmetric or anti-symmetric boundary conditions (depending on the dipole orientation) at the sides. The electric field was recorded on a set of screens surrounding the lens at the top and the sides, and a built-in *farfieldexact* function was used to calculate the far-field light intensity for the three cardinal dipole orientations. Their incoherent sum represented the total far-field radiation distribution. It was then integrated over the emission angle and normalised to give the collected power of the emitted light as a function of the collection half-angle. The simulations were also carried out for a half-spherical lens and a bare substrate.

Ray-tracing simulations were performed with custom software written in Python 3.7 featuring the full form of the Fresnel equations and taking into account recursive reflections at the interfaces between different materials in the 3D simulation box.

## Supplementary information

Supplementary information for ″Ultra-long-working-distance spectroscopy of single nanostructures with aspherical solid immersion microlenses″.

## Data Availability

The data that support the findings of this study for modelling are available from the corresponding author on reasonable request.

## Code availability

The code for modelling is available from the corresponding author on reasonable request.
