# Supplementary material for: Ultra-long-working-distance spectroscopy of single nanostructures with aspherical solid immersion microlenses
Source: Light Sci Appl. 2020 Mar 27;9:48. doi: 10.1038/s41377-020-0284-1 (PMC7101340; doi:10.1038/s41377-020-0284-1)
Supplement: Supplementary file 1 — Supplementary information for ″Ultra-long-working-distance spectroscopy of single nanostructures with aspherical solid immersion microlenses″. [file 41377_2020_284_MOESM1_ESM.pdf]

Ultra-long-working-distance spectroscopy of single nanostructures with aspherical solid immersion microlenses - supplementary information

Aleksander Bogucki*,[1] Łukasz Zinkiewicz,[1] Magdalena Grzeszczyk,[1] Wojciech Pacuski,[1] Karol Nogajewski,[1] Tomasz Kazimierczuk,[1] Aleksander Rodek,[1] Jan Suffczyński,[1] Kenji Watanabe,[2] Takashi Taniguchi,[2] Piotr Wasylczyk,[1] Marek Potemski,[1,3] and Piotr Kossacki†[1]

[1] *Faculty of Physics, University of Warsaw, ul. Pasteura 5, 02-093 Warsaw, Poland*
[2]*National Institute for Materials Science, Tsukuba, Ibaraki, 305-0044, Japan*
[3]*Laboratoire National des Champs Magnétiques Intenses, CNRS-UJF-UPS-INSA, 25, avenue des Martyrs, 38042 Grenoble, France*

This supplementary information contains:

- Supplementary Fig. S1: Design and scalability of the µ-lenses.
- Supplementary Fig. S2: The optimal shape analysis of the µ-lenses.
- Supplementary Fig. S3: Coupling an optical fibre with the µ-lens.
- Supplementary Fig. S4: Direct Laser Writing (DLW) of µ-lenses on semiconducting transition metal dichalcogenide (S-TMD) heterostructures.
- Supplementary Fig. S5: Low-temperature photoluminescence spectrum of an hBN/$MoSe_2$ heterostructure equipped with the µ-lens.
- Supplementary Fig. S6: Photoluminescence spectrum of the two-photon polymerised IP-Dip resist used for 3D printing of the µ-lenses.
- Supplementary Fig. S7: Raman scattering spectrum of two-photon polymerised (TPP) IP-Dip resist used for 3D printing of the µ-lenses.
- Supplementary Fig. S8: Height profiles of thin layers of two-photon polymerised (TPP) IP-Dip resist used for characterisation measurements.
- Supplementary Fig. S9: Influence of temperature on transmission properties of an IP-Dip resist array polymerised by two-photon absorption.
- Supplementary Fig. S10: Absorption of an IP-Dip resist array fabricated by two-photon polymerisation (TPP).
- Supplementary Equation E1: Spin Hamiltonian used in the calculation of the QDs spectra presented in Fig. 4g of the main text.
- Supplementary Table T1: Model Hamiltonian parameters used in the calculation of the QDs spectra presented in Fig. 4g of the main text.

* aleksander.bogucki@fuw.edu.pl
† piotr.kossacki@fuw.edu.pl

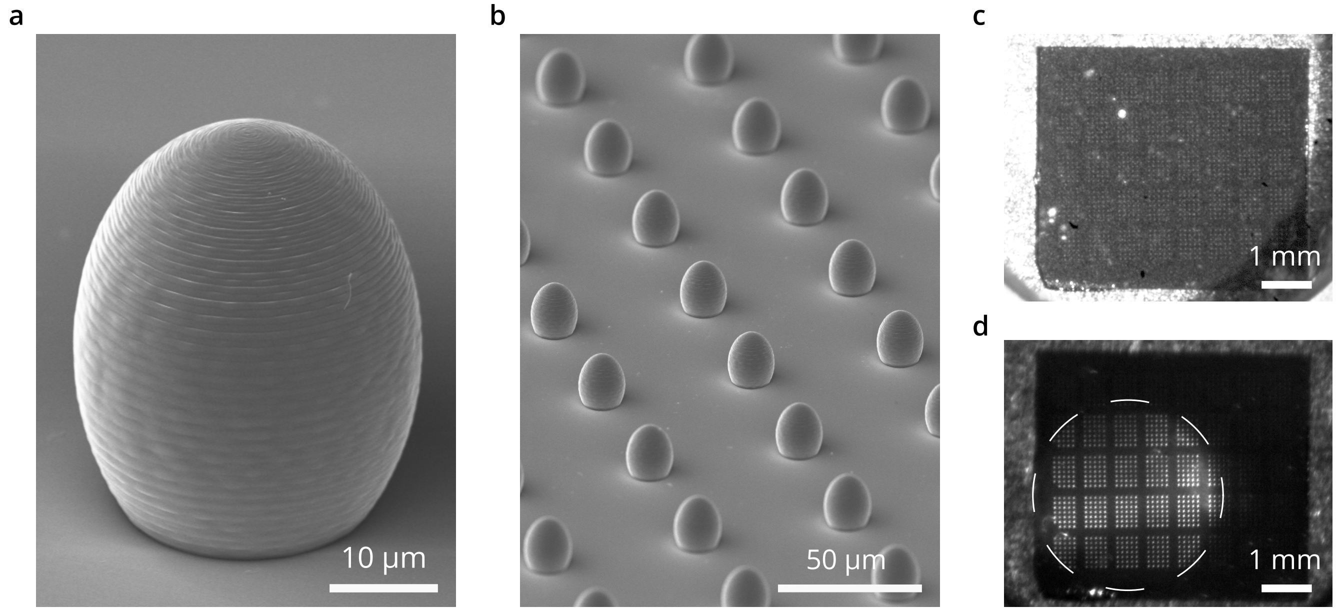

Supplementary Fig. S1: Design and scalability of the μ-lenses. During the fabrication of μ-lenses we used a few techniques to improve the quality of the lens surface while maintaining a short printing time [1]. Due to axial symmetry of the μ-lens, each z-layer along the symmetry axis was exposed along a spiral path (annular scanning method). The shell of the μ-lens was printed with a multi-path scanning technique - the outer circle was exposed three times with 80% of the regular laser power and with increased angular accuracy. We also used adaptive slicing in the z direction - the distance between the layers was smaller for the top regions of the μ-lens. These techniques resulted in a relatively smooth surface (**a**) and printing time of about 3 minutes for a μ-lens with 7.5 μm radius. This allowed us to efficiently print hundreds of μ-lens over large-area samples (**b**) and eliminated the low-temperature photoluminescence characterisation of the quantum dots during or before the fabrication of the μ-lenses. Image (**c**) shows 40 fields of μ-lenses with 35 μ-lenses in each field, giving in total 1400 μ-lenses printed over an area of 6 mm $\times$ 4.8 mm. The contrast of the image was adjusted to increase the visibility of light scattered from the μ-lenses. An array of μ-lenses allows for easy identification of a desired μ-lens with an emitter of interest underneath as well as for finding a particular one in a different experimental setup or at a different time. Panel (**d**) shows a preview image of a sample illuminated by a defocused laser beam (excitation area is marked by the dashed circle) after filtering the laser light with a long-pass filter. The photoluminescence from the μ-lenses is visible as bright spots. The images (**c,d**) were obtained with a standard preview setup consisting of a white light illumination source, a beam splitter and a CMOS camera equipped with a f=180 mm objective lens.

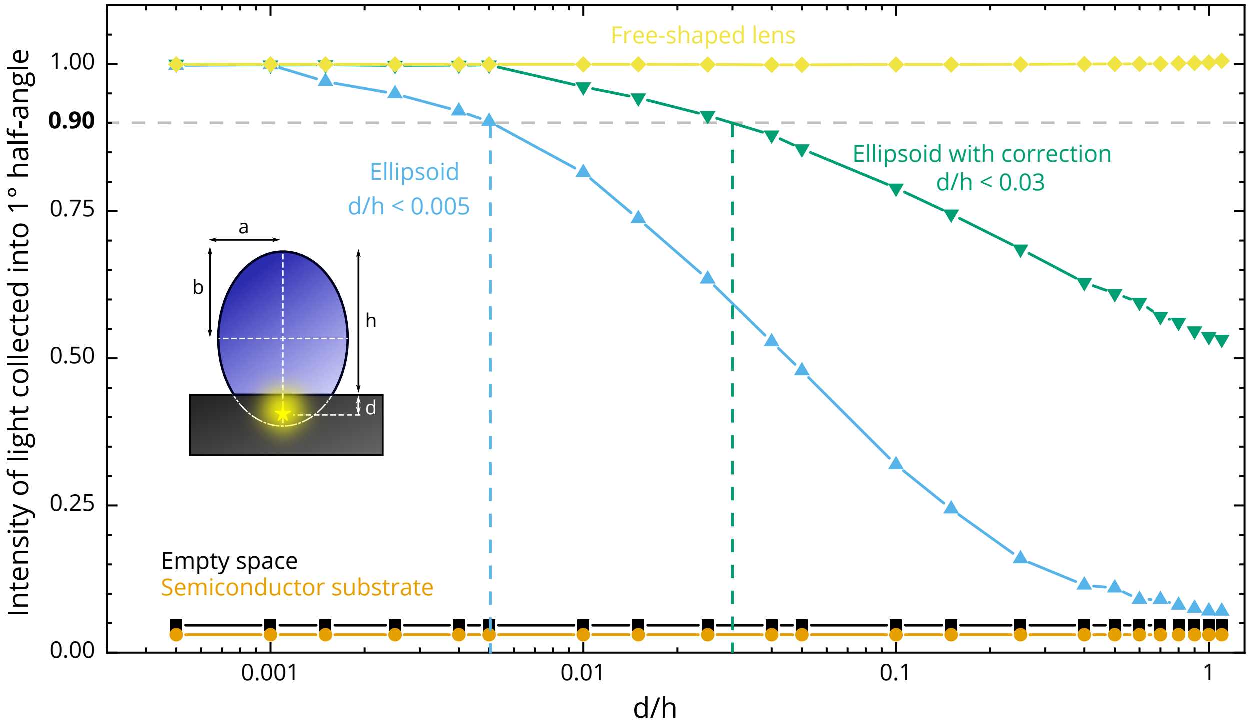

Supplementary Fig. S2: The optimal shape analysis of the μ-lenses. The distance to the surface d of an emitter embedded in a semiconductor substrate influences the optimal shape of a μ-lens with fixed height h. We calculated the amount of light collected into a half-angle of 1° for a free-shape lens, an elliptical lens, and an elliptical lens with a correction of refraction based on Snell's law (see main text) as a function of the d/h ratio. The plot shows the result of a ray-tracing simulation normalised to the amount of light obtained for the optimal free-shaped lens. The d/h ratio for a typical QD emitter located 50 nm below the surface of a high refractive index semiconductor (n=3.01) and a μ-lens made of resist (n=1.53) with fixed height of h=20 μm equals 0.0025. This shows that for most practical realisations where a point emitter is embedded in a high-refractive index substrate, the ellipsoidal shape allows for collecting more than 90% of emitted light. Taking into account the analytic correction for propagation of light in the substrate improves this performance even more. The simulations were performed for the wavelength $\lambda = 635$ nm with a custom ray-tracing software written in Python 3.7 scripting language.

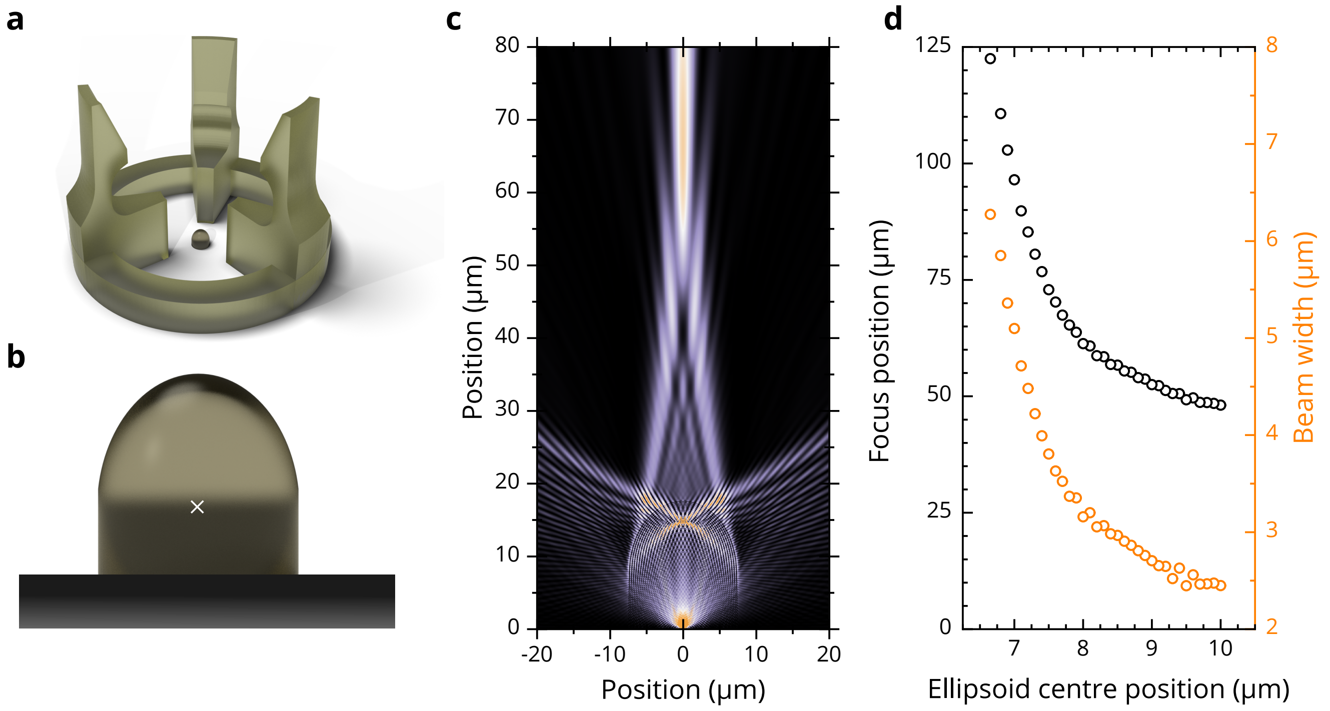

Supplementary Fig. S3: Coupling an optical fibre with the μ-lens. The μ-lenses are also well-suited for focusing photoluminescence beam at short distances - e.g. for efficient introduction into an optical fibre. (**a**) Visualisation of an ellipsoidal lens placed inside a DLW-printed micro fibre port allowing for precise fibre positioning. The port design is similar to the one presented in [2]. (**b**) The only modification to the μ-lens parameters is shifting its centre (denoted with the white "×" mark) away from the substrate surface while keeping the same radii as those calculated for for the case of light collimation. Additional cylindrical filling is added into the volume between the lens and the substrate. (**c**) Light intensity map obtained for the μ-lens centre placed 7.7 μm above the substrate surface calculated with Finite Difference Time-Domain simulations (FDTD Solutions, Lumerical Inc.) for a monochromatic ($\lambda = 633$ nm) point source located 50 nm below the substrate surface. The light is focused approximately 70 μm away from the substrate and the ellipsoidal shape of the μ-lens is visible in the lower part of the map. (**d**) A calculated focus position with respect to the substrate surface (black) and the full beam width at $1/e^2$ of its peak intensity in the focus (orange) as a function of the ellipsoid centre position above the substrate. The beam widths match well the typical mode field diameters of single mode fibres while the minimum port size, determined by the focal spot distance to the substrate, is still in the range of standard DLW-system capabilities.

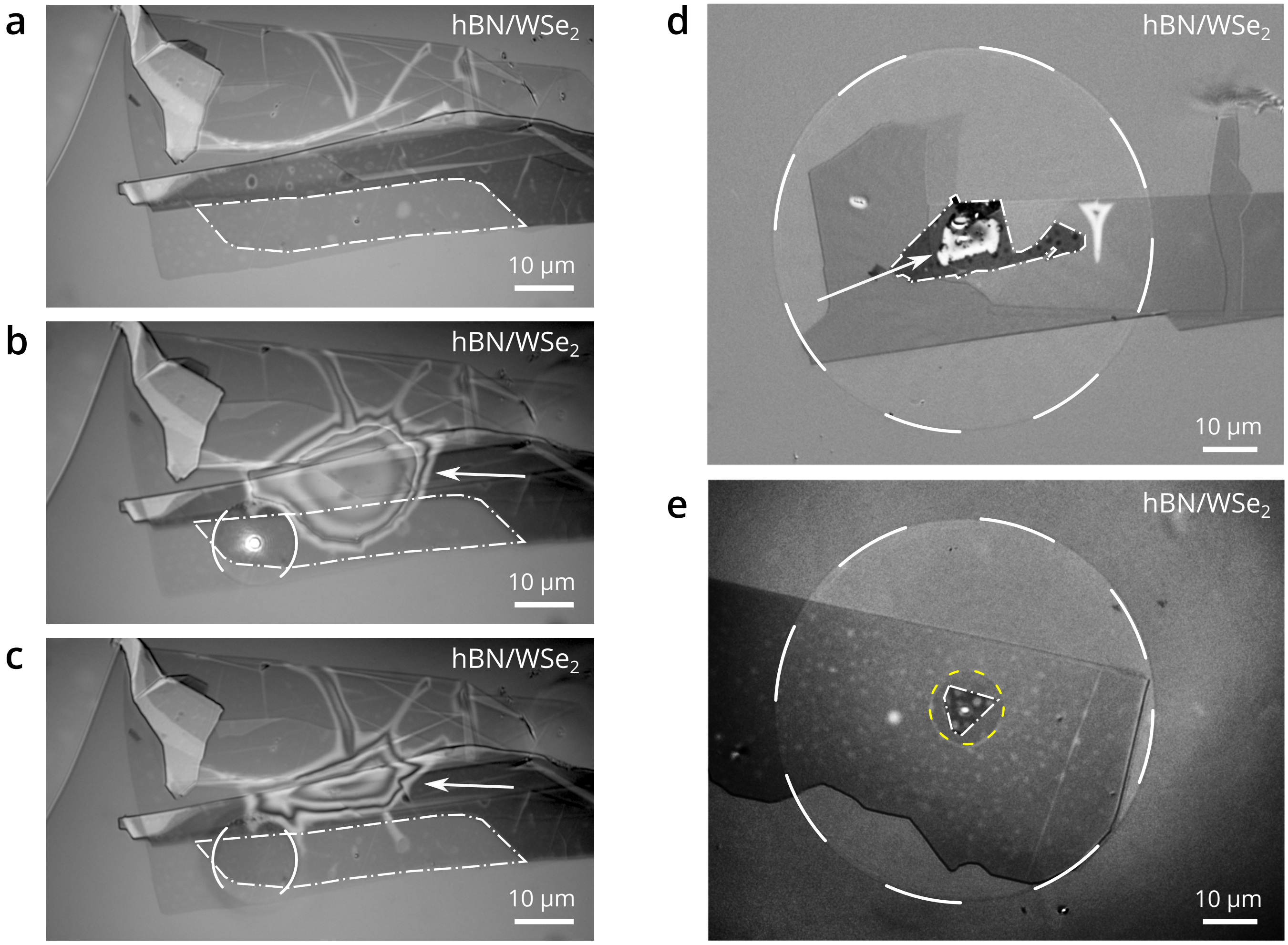

Supplementary Fig. S4: Direct Laser Writing (DLW) of μ-lenses on semiconducting transition metal dichalcogenide (S-TMD) heterostructures. One of the risks encountered during printing μ-lenses on S-TMD heterostructures is related to imperfections located at the sample interface. These can lead to delamination of the heterostructure or formation of bubbles in the resist during laser exposure. (**a**) Optical image of an hBN/WSe_2 heterostructure before printing a 15-μm in diameter μ-lens. The WSe_2 monolayer is marked with the white dash-dot polygon. The proximity of uneven surfaces of a thicker part of the WSe_2 flake may decrease the adhesion between the WSe_2 monolayer and the hBN protective cover. (**b**) Interference pattern between the bottom S-TMD and the top hBN layers appearing during the printing procedure. The two materials are not in contact, but separated by a cavity marked with the white arrow. The outline of the μ-lens (dashed white circle) with a bright lithographic laser spot in the middle is visible in the bottom left. (**c**) After printing, the cavity size decreases due to adhesion between the S-TMD and hBN flakes. In the upper part of the printed μ-lens there are dark spots - inclusions formed from overheated resist. (**d**) Image captured after printing a 1 μm high layer of a 70-μm in diameter μ-lens (white dashed circle). The WSe_2 monolayer (dash-dot polygon) is covered with an hBN flake consisting of a few regions with different thicknesses. In the middle part of the WSe_2 monolayer a bright bubble formed in the resist is visible (marked with the white arrow). (**e**) Image captured after printing a 1 μm high layer of a 70-μm in diameter μ-lens (white dashed circle) over another WSe_2 monolayer (white dash-dotted polygon). To avoid bubble formation a 12-μm in diameter and 1 μm high cylinder, located at the centre of a μ-lens, is left unexposed (yellow dashed circle). One-photon exposure of this part can be done with UV light after the development of the lens. The above remarks do also apply to $MoSe_2$-based heterostructures. (**a-e**) All images were captured during the fabrication of the μ-lenses with Photonic Professional (Nanoscribe GmbH) 3D photolithography system with a ×100 NA=1.3 immersion microscope objective (Zeiss GmbH) and IP-Dip resist (Nanoscribe GmbH), being also an immersion medium.

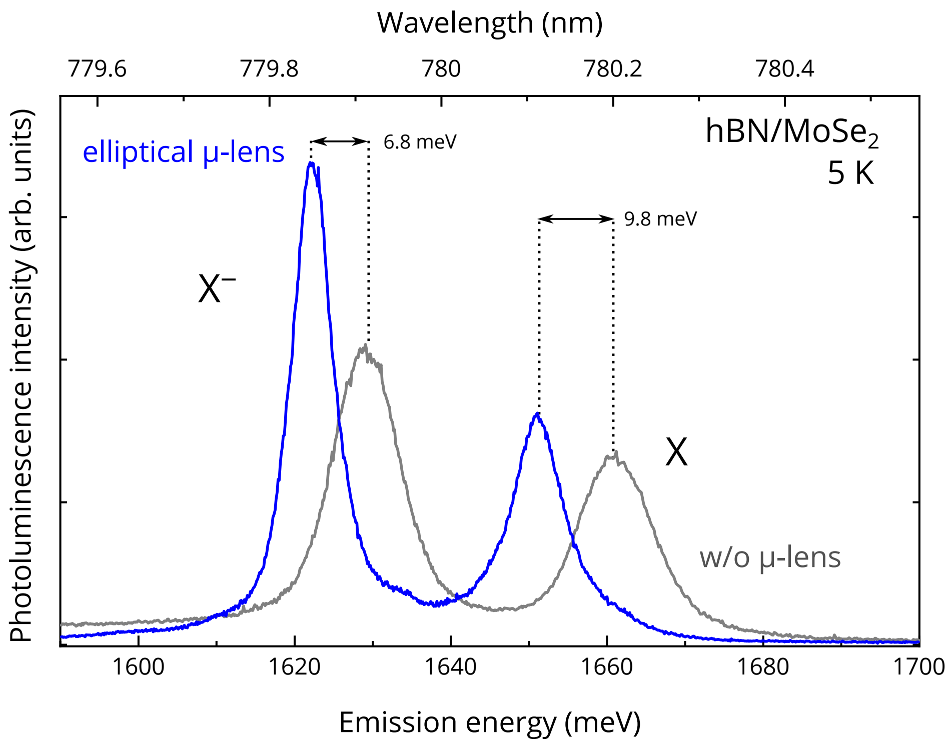

Supplementary Fig. S5: Low-temperature photoluminescence spectrum of an hBN/MoSe$_2$ heterostructure equipped with the μ-lens. After 3D printing of the μ-lens, we observed a narrowing of the low-temperature (5 K) PL emission lines as well as shifting of the peaks towards lower energies (blue curve), as compared to the spectrum acquired without the μ-lens (grey curve). We attribute the energy shift to the strain introduced by the printed structure. According to the available data [3–6] the strain corresponding to the above peak shifts should be approximately 0.4%. The fitted peaks parameters are: E^{X}_{before} = (1660.9 ± 0.2) meV, w^{X}_{before} = (13.02 ± 0.12) meV, $E^{X^-}_{before}$ = (1629.06 ± 0.02) meV, $w^{X^-}_{before}$ = (12.07 ± 0.14) meV ; E^{X}_{after} = (1651.09 ± 0.01) meV, w^{X}_{after} = (8.25 ± 0.05) meV, $E^{X^-}_{after}$ = (1622.26 ± 0.01) meV, $w^{X^-}_{after}$ = (6.43 ± 0.08) meV, where E stands for the peak position and w for its width. Subscript *before* corresponds to values measured without μ-lens, whereas subscript *after* corresponds to values measured after printing the μ-lens.

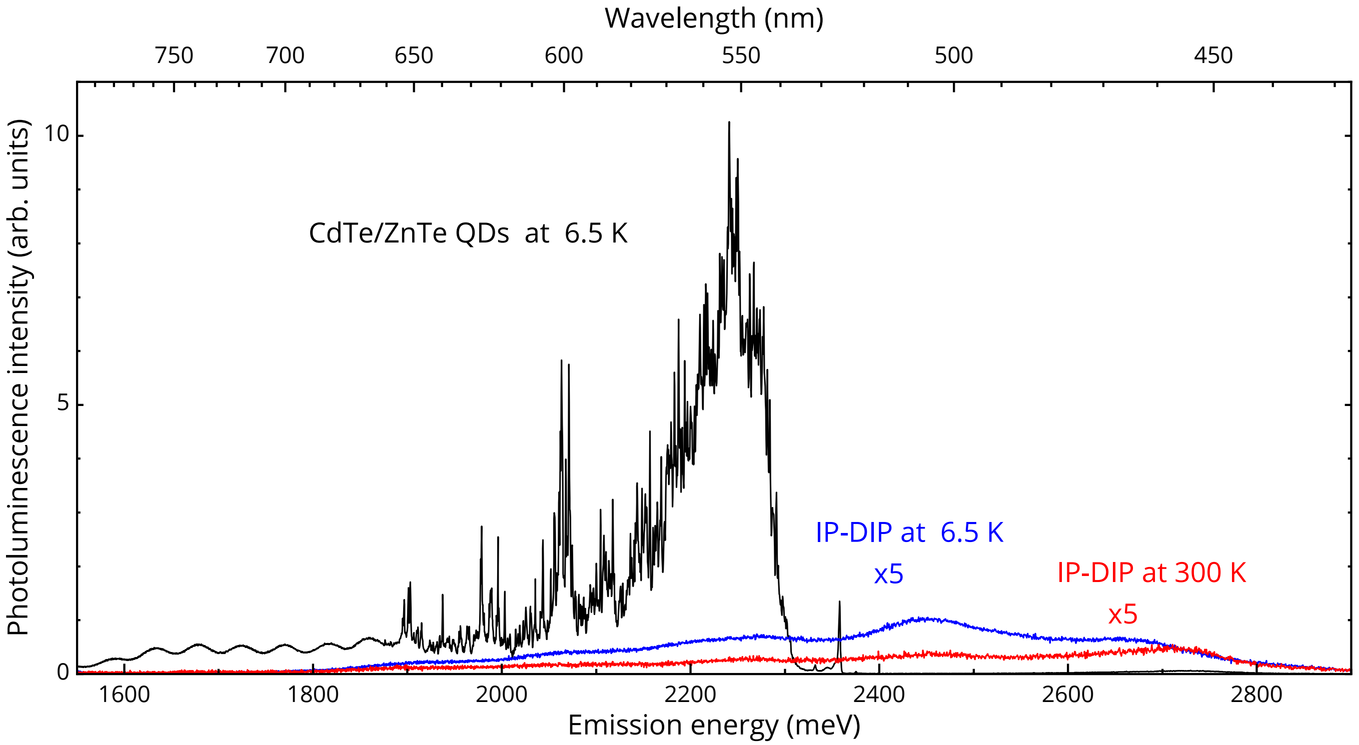

Supplementary Fig. S6: Photoluminescence spectrum of the two-photon polymerised (TPP) IP-Dip resist used for 3D printing of the μ-lenses. We measured the photoluminescence of two-photon polymerised (TPP) IP-Dip (Nanoscribe GmbH) negative-tone photoresist at 6.5 K (blue curve) and at 300 K (red curve) under excitation of a blue (λ = 405 nm) laser. Additionally, at 6.5 K and in the same experimental setup we collected also the photoluminescence signal from CdTe/ZnTe quantum dots. The photoluminescence intensity of the TPP IP-Dip resist is over 50 times weaker than the signal from typical quantum dots. Moreover, the photoluminescence signal of the photoresist vanishes below 675 nm. This shows that the TPP IP-Dip resist can be used at both room and cryogenic temperatures.

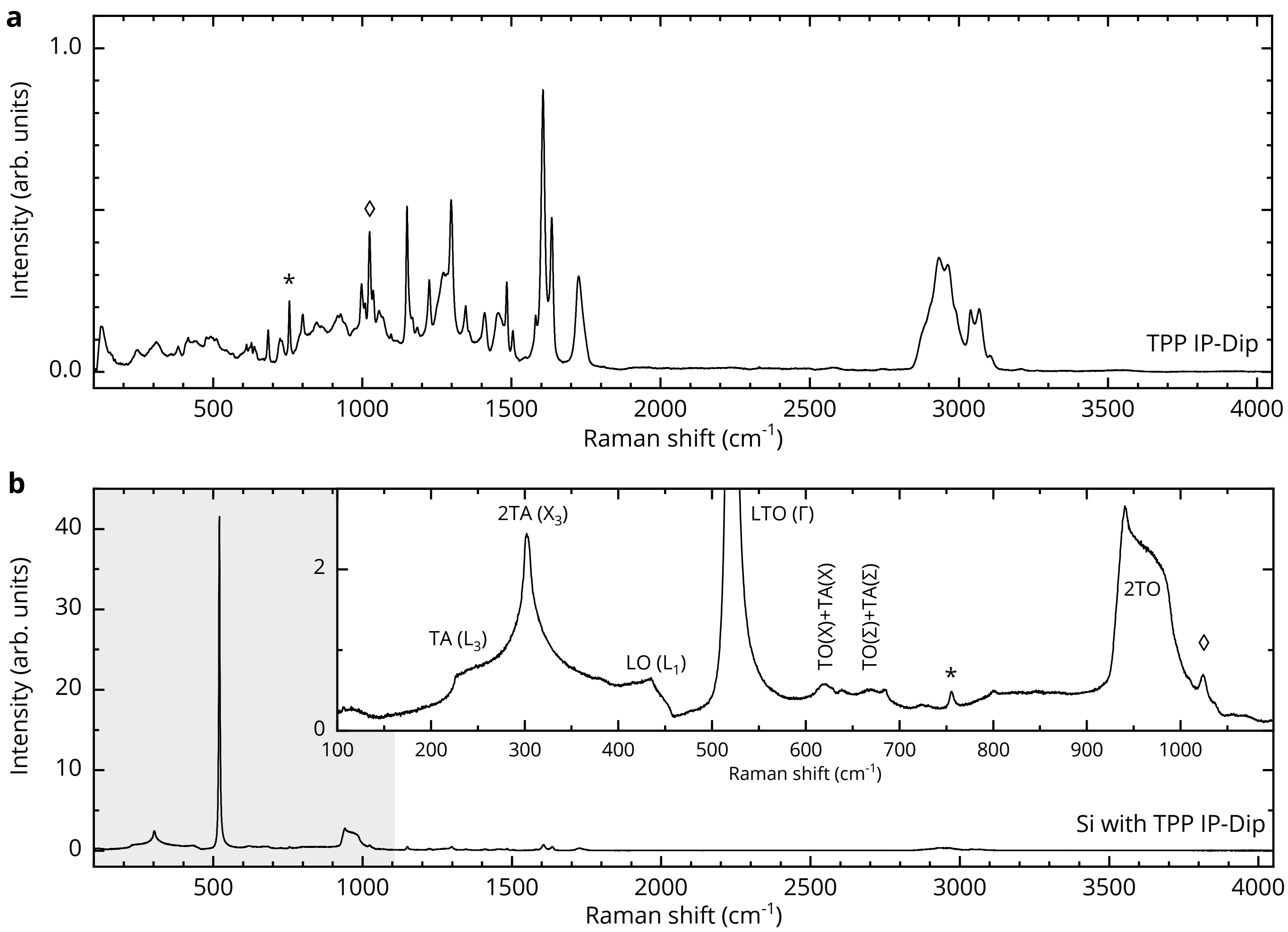

Supplementary Fig. S7: Raman scattering spectrum of two-photon polymerised (TPP) IP-Dip resist used for 3D printing of the μ-lenses. (**a**) Raman scattering spectrum of the two-photon polymerised (TPP) IP-Dip (Nanoscribe GmbH) negative-tone photoresist measured with a $\lambda = 633$ nm laser at room temperature. (**b**) Raman scattering spectrum of a silicon substrate covered with a 5 μm thick layer of the TPP IP-Dip photoresist measured under the same conditions. The spectrum is dominated by a strong peak at 520 cm^{-1} associated with the LTO silicon mode. The intensity of this peak is approximately 80 times stronger than the signal from the most pronounced line of the IP-Dip resist. The inset shows a magnification of the shadowed area in the lower panel. The peaks visible in the inset can also be attributed to different silicon modes. Two peaks which do not originate from the silicon substrate can be assigned to the resist and are marked in both panels by the asterisk ($\star$) and diamond ($\diamond$). Therefore, the TPP IP-Dip resist is suitable for a wide variety of Raman spectroscopy measurements.

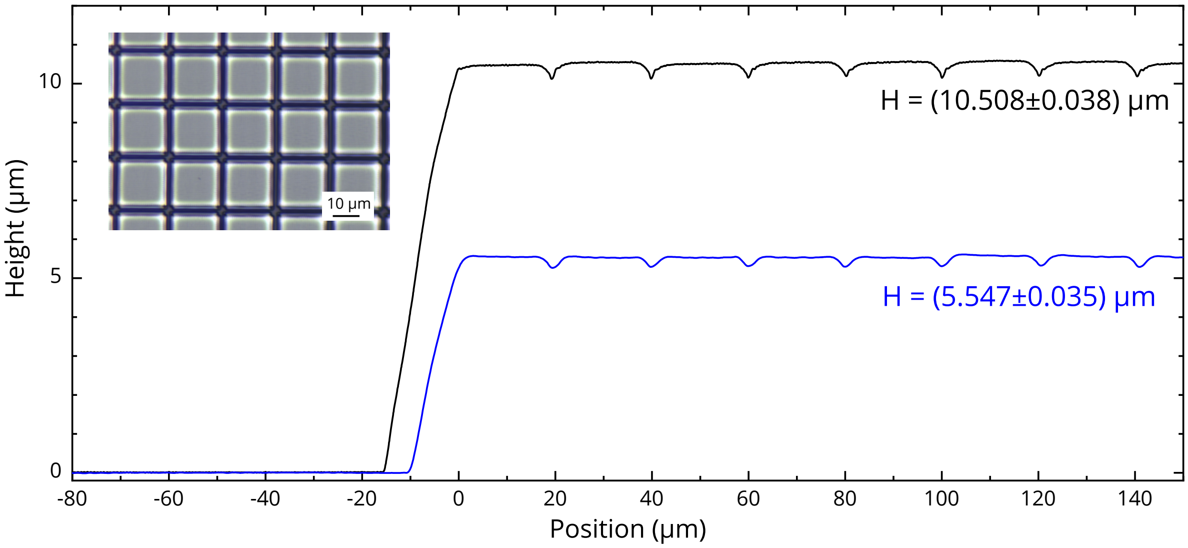

Supplementary Fig. S8: Height profiles of thin layers of two-photon polymerised (TPP) IP-Dip resist used for characterisation measurements. To measure the absorption coefficient of the resist, two macroscopic layers with lateral dimensions of 1120 µm × 1120 µm and thicknesses differing by approximately a factor of 2 were two-photon polymerised on a piece of glass. The entire photoresist areas consisted of an array of smaller tiles with the one-tile unit being a 19 µm × 19 µm block surrounded by a 1 µm gap as shown in the inset. This helped to avoid detaching of the polymerised structure from the substrate due to the resist shrinkage [7]. The heights of the printed structures were measured with Vecco Dectak 150 Surface Profiler using a high-aspect-ratio 10 µm × 2 µm stylus. The measured heights of thinner and thicker layer are (5.547 ± 0.035) µm and (10.508 ± 0.038) µm, respectively.

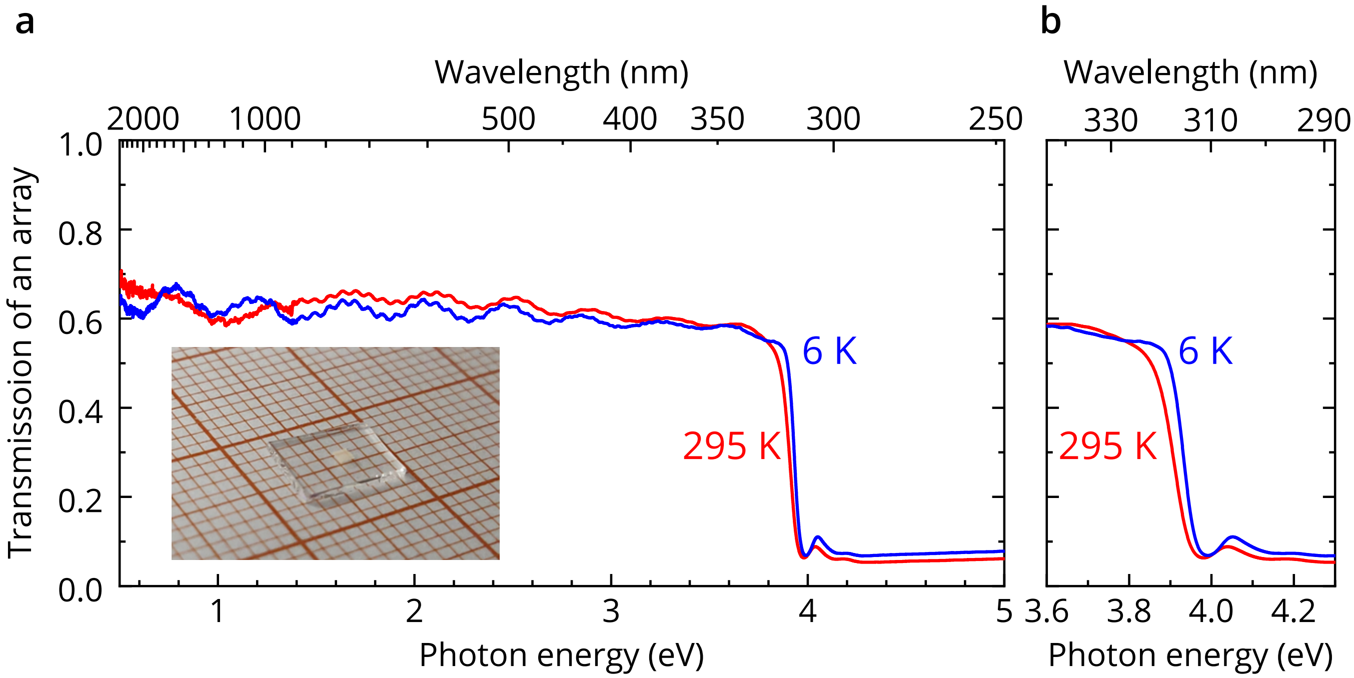

Supplementary Fig. S9: Influence of temperature on transmission properties of an IP-Dip resist array polymerised by two-photon absorption. (**a**) We measured the transmission coefficients for a 5 μm thick layer of the IP-Dip photoresist (see Supplementary Fig. S8) at room (295 K) and cryogenic (6 K) temperatures using Varian Cary 5000 UV/VIS/NIR spectrometer. The transmission is reduced due to light scattering on the tile-structured array (as visible also with a naked eye – see the inset). (**b**) A 13 meV shift of the absorption edge towards higher energies is observed when the temperature is lowered from 295 K to 6 K. Besides the blueshift of the absorption edge there are no other significant changes in the transmission spectrum which shows that the IP-Dip (Nanoscribe GmbH) resist is well-suited for cryogenic-temperature applications.

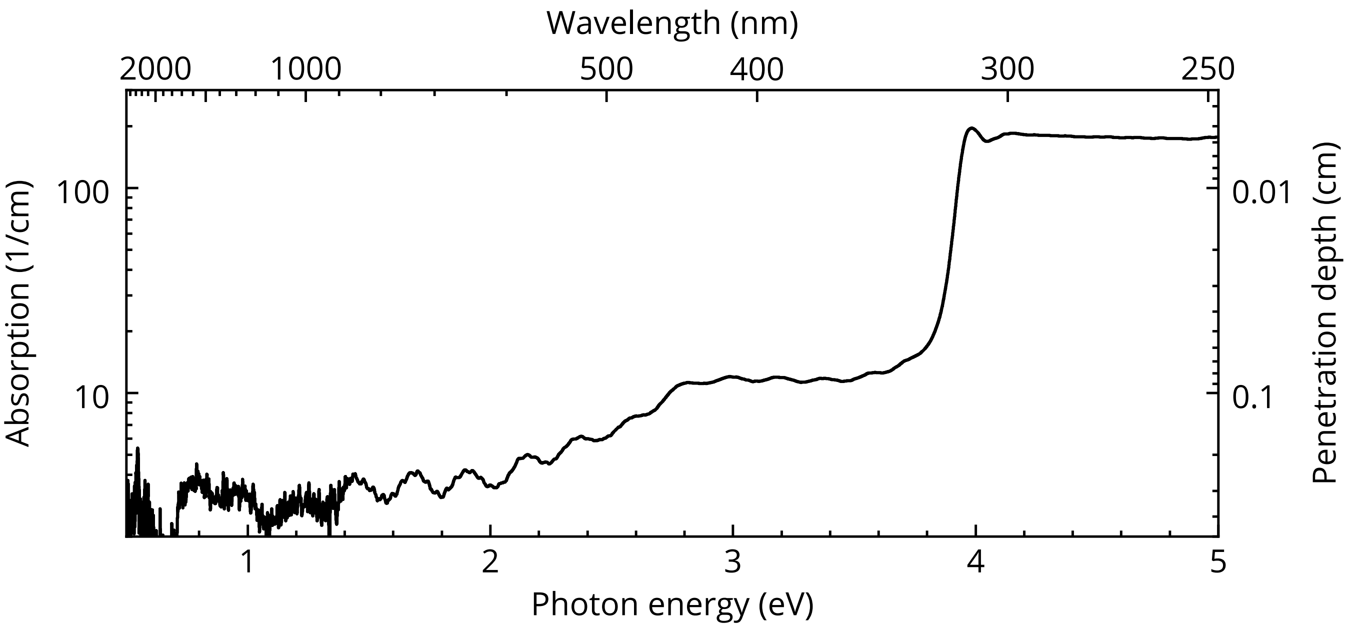

Supplementary Fig. S10: Absorption of an IP-Dip resist array fabricated by two-photon polymerisation (TPP). (**a**) An absorption coefficient of the IP-Dip photoresist was calculated based on room-temperature transmission measurements done on the 5 µm and 10 µm thick photoresist layers (see Supplementary Fig. S8). The measurements were performed using Varian Cary 5000 UV/VIS/NIR spectrometer. The spectrum shown indicates that the IP-Dip (Nanoscribe GmbH) resist is suitable for optical applications at energies lower than 3.6 eV (345 nm). We have checked that the absorption edge of the IP-Dip resist polymerised by single-photon absorption is shifted to lower energies by about 0.2 eV (not shown) with respect to that of the IP-Dip resist polymerised by two-photon absorption.

Supplementary Eq. E1: Spin Hamiltonian used in the calculation of the QDs spectra presented in Fig. 4g of the main text. To simulate the magnetic-field evolution of the neutral exciton emission line we used a standard Hamiltonian [8, 9] with the input parameters provided in Supplementary Table S1.

$$\hat{H} = \frac{1}{2}\begin{bmatrix} \Delta_{\mathrm{s,p-d}} & \delta_1 & \delta_{\mathrm{Fe}} & 0 \\ \delta_1 & -\Delta_{\mathrm{s,p-d}} & 0 & \delta_1 \\ \delta_{\mathrm{Fe}} & 0 & -\Delta_{\mathrm{s,p-d}} & \delta_{\mathrm{Fe}} \\ 0 & \delta_1 & \delta_{\mathrm{Fe}} & \Delta_{\mathrm{s,p-d}} \end{bmatrix} + \mu_{\mathrm{B}} g_{\mathrm{X}} S_X^z B + \mu_{\mathrm{B}} g_{\mathrm{Fe}} S_{\mathrm{Fe}}^z B + \gamma B^2, \quad (1)$$

Supplementary Tab. T1: Model Hamiltonian parameters used in the calculation of the QDs spectra presented in Fig. 4g of the main text. The meaning of particular parameters is explained elsewhere [8–10].

**Parameter**	$\Delta_{\mathrm{s,p\text{-}d}}$ (meV)	δ_1 (meV)	δ_{Fe} (meV)	g_{X}	g_{Fe}	γ (meV/T^2)	T (K)
**Value**	0.25	0.47	<0.05	1.38	2	0.001	30

[1] X. Zhou, Y. Hou, and J. Lin, “A review on the processing accuracy of two-photon polymerization”, AIP Adv. **5**, 030701 (2015).

[2] A. Bogucki, Ł. Zinkiewicz, W. Pacuski, P. Wasylczyk, and P. Kossacki, “Optical fiber micro-connector with nanometer positioning precision for rapid prototyping of photonic devices”, Opt. Express **26**, 11513 (2018).

[3] L. Mennel, M. Paur, and T. Mueller, “Second harmonic generation in strained transition metal dichalcogenide monolayers: MoS_2, $MoSe_2$, WS_2, and WSe_2”, APL Photonics **4**, 034404 (2018).

[4] R. Frisenda, M. Drüppel, R. Schmidt, S. M. de Vasconcellos, D. P. de Lara, R. Bratschitsch, M. Rohlfing, and A. Castellanos-Gomez, “Biaxial strain tuning of the optical properties of single-layer transition metal dichalcogenides”, NPJ 2D Mater. Appl. **1**, 10 (2017).

[5] J. O. Island, A. Kuc, E. H. Diependaal, R. Bratschitsch, H. S. J. van der Zant, T. Heine, and A. Castellanos-Gomez, “Precise and reversible band gap tuning in single-layer $MoSe_2$ by uniaxial strain”, Nanoscale **8**, 2589–2593 (2016).

[6] J. Ji, A. Zhang, T. Xia, P. Gao, Y. Jie, Q. Zhang, and Q. Zhang, “Strain-modulated excitonic gaps in mono- and bi-layer $MoSe_2$”, Chin. Phys. B **25**, 077802 (2016).

[7] Y. Liu, J. H. Campbell, O. Stein, L. Jiang, J. Hund, and Y. Lu, “Deformation Behavior of Foam Laser Targets Fabricated by Two-Photon Polymerization”, Nanomaterials **8**, 498 (2018).

[8] T. Smoleński, T. Kazimierczuk, J. Kobak, M. Goryca, A. Golnik, P. Kossacki, and W. Pacuski, “Magnetic ground state of an individual Fe^{2+} ion in strained semiconductor nanostructure”, Nat. Commun. **7**, 10484 (2016).

[9] A. Rodek, T. Kazimierczuk, A. Bogucki, T. Smoleński, W. Pacuski, and P. Kossacki, “Readout of a dopant spin in the anisotropic quantum dot with a single magnetic ion”, J. Phys. Condens. Matter **31**, 455301 (2019).

[10] J. Kobak, T. Smoleński, M. Goryca, M. Papaj, K. Gietka, A. Bogucki, M. Koperski, J.-G. Rousset, J. Suffczyński, E. Janik, M. Nawrocki, A. Golnik, P. Kossacki, and W. Pacuski, “Designing quantum dots for solotronics”, Nat. Commun. **5**, 3191 (2014).
